# Studies on the Antiliver Injury of Effective Parts from *Saururus chinensis* by Rats and Mice Model *In Vivo*

**DOI:** 10.1155/2022/7821724

**Published:** 2022-04-26

**Authors:** Chaonan Wang, Dongyan Cheng, Mingming Yan, Lishu Wang

**Affiliations:** ^1^Changchun University of Chinese Medicine, Changchun 130117, China; ^2^Jilin Provincial Academy of Chinese Medicine Sciences, Changchun 130021, China

## Abstract

The aim of this study was to evaluate the pharmacodynamics of the effective parts from *Saururus chinensis* (EPS) *in vivo*. The antihepatic fibrosis and injury effects of EPS were investigated with the following four model animals including the effect on Wistar rats with liver fibrosis induced by complex factors, mice with acute liver injury induced, respectively, by carbon tetrachloride and alcohol, and Sprague-Dawley (SD) rats with acute liver injury caused by D-galactosamine hydrochloride. The pharmacodynamics results showed that the rats' oral administration of EPS can significantly inhibit the formation of liver fibrosis in rats caused by complex factors and has significant preventive and therapeutic effects on acute liver injury caused by various factors as shown by decreased levels of serum biochemical indicators and improved pathological grade. Taken all together, our findings showed that EPS exhibits potent activities and should be considered a good option and an additional source of natural compounds for the treatment of hepatic fibrosis and hepatic injury.

## 1. Introduction

*Saururus chinensis* (*S*. *chinensis*) is the dry aerial part of *Saururus chinensis* (Lour.) Baill (*saururaceae*), and it has been used in chinese folk medicine for the treatment of various diseases such as jaundice, urinary tract infection, edema of nephritis, dysuria, gonorrhea, leucorrhea abnormality, swelling sores, and eczema [1, 2]. Modern studies have shown that *S*. *chinensis* contains lignans, alkaloids, terpenes, flavonoids, and other components and has a variety of biological activities such as anti-inflammatory, antitumor, and antioxidative [3].

Liver fibrosis is the intermediate link and necessary stage of various causes of liver disease from the progressive development to end-stage liver disease, and it is very important to prevent the progression of liver fibrosis and the reversion of developed liver fibrosis, which can reduce the occurrence of end-stage liver disease (cirrhosis, liver cancer) and thus reduce the mortality related to liver disease. It is urgent to reduce the damage to the liver and to slow down the progress of liver injury to fibrosis and cancer.

Therefore, on the basis of our previous chemical components and biological studies of *S*. *chinensis* [4–9], in our present study, effective parts from the herbs of *S*. *chinensis* (EPS) (the sum of total lignans, total lactams, and total sterols is greater than 50%) were used to examine its hepatoprotective effects. Its antiliver injury efficacy was studied systematically by four animal models, including the effects on liver fibrosis rats induced by complex factors, acute liver injury mice and rats caused by carbon tetrachloride, alcohol, and D-galactosamine hydrochloride, respectively. The liver inflammation and hepatofibrosis-improving effects were evaluated according to AST, ALT (serum biochemical indicators for liver inflammation), HA, LN, and COLIV, PC III (four serum markers of rats with liver fibrosis), TP, ALB (liver cell regeneration indicators), SOD, T-AOC, and MDA (antioxidant indicators), as well as histopathological examination of rats and mice liver. This is the first study systematically on hepatoprotective effects of *S*. *chinensis in vivo*. This is the first time to use four different liver injury models to study antifibrosis and liver injury systematically.

## 2. Materials and Methods

### 2.1. Plant Material

The whole plant materials were purchased and collected from Chenzhou City, Hunan Province, and identified as *S*. *chinensis* by Professor Dacheng Jiang, College of Pharmacy, Changchun University of Chinese Medicine, China.

### 2.2. Preparation of Experimental Medication (EPS)

The dried herbs of *S*. *chinensis* (10 kg) were cut into sections, extracted twice with aqueous 70% EtOH under reflux for 2 hours and 8 times the amount of medicinal material each time. The extract was concentrated until the weight ratio (EtOH-dried herb) was 1 : 1, left to stand at room temperature overnight, brown yellow precipitates were obtained after filtration of the solution, the precipitates were further purified and dried at 55°C by vacuum drying, and the effective parts of *S*. *chinensis* (EPS) were obtained (232 g).

### 2.3. Medication Composition Analysis

The content of total lignans and lactams in EPS was determined by UV spectrophotometry, and total sterols were determined by vanillin-perchloric acid method [8–10]. The determinations process is as follows.

#### 2.3.1. Content Determinations of Total Lignans

0.1 mL, 0.2 mL, 0.3 mL, 0.4 mL, 0.5 mL, 0.6 mL, and 0.7 mL of the reference solution (0.25 mg Sauchinone per mL) were accurately measured, the absorbance was measured at the wavelength of 242 nm by UV-Vis spectrophotometry, the standard curve with absorbance was drawn, and about 25 mg of EPS powder was accurately weighed, put into a conical flask, added 40 mL of chloroform, ultrasonic treated (Power 120 W, frequency 40 KHz) for 20 minutes, filtered, quantitatively transferred the filtrate into a 50 mL measuring flask, added chloroform to the scale, and shaken well. 0.6 mL of the sample solution was accurately measured, put into a 10 mL measuring bottle, added chloroform to the scale, and shaken well. With the corresponding reagent as the blank, the absorbance was measured at the wavelength of 242 nm by UV-Vis spectrophotometry. The concentration of total lignans in the test solution was calculated with the standard curve.

#### 2.3.2. Content Determinations of Total Lactams

0.3 mL, 0.6 mL, 0.9 mL, 1.2 mL, 1.5 mL, and 1.8 mL of the sauristolactam reference solution (90 *μ*g/mL) were accurately measured, the absorbance was measured at the wavelength of 277 nm by UV-Vis spectrophotometry, the standard curve with absorbance was drawn, and about 13 mg of EPS powder was weighed accurately, placed in a conical flask with a stopper, accurately added 25 mL of methanol, weighed, ultrasonic treated (Power 120 W, frequency 40 KHz) for 20 minutes, cooled, weighed again, 0.8 mL of supernatant was accurately measured, put into a 10 mL measuring bottle, methanol was added to the scale, and shaken well. With the corresponding reagent as the blank, the absorbance was measured at the wavelength of 277 nm by UV-Vis spectrophotometry. The concentration of total lactams was calculated in the test solution with the standard curve.

#### 2.3.3. Content Determinations of Total Sterols

The *β*-sitosterol reference solution of 25 *μ*L, 50 *μ*L, 75 *μ*L, 100 *μ*L, 125 *μ*L, and 150 *μ*L (0.6 mg per mL) was accurately measured, put into the test tube with stopper, respectively, volatilized the solvent, added 0.2 mL of newly prepared 5% vanillin glacial acetic acid solution and 0.8 mL of perchloric acid solution, shaken well, kept warm in a water bath at 60°C for 15 minutes, cooled with running water, added 5 mL of glacial acetic acid, and shaken well. With the corresponding reagent as the blank, the absorbance was measured at the wavelength of 541 nm by UV-Vis spectrophotometry, and about 25 mg of this product powder was weighed accurately, put into a conical flask, added 30 mL of chloroform, ultrasonic treated (power 120 W, frequency 40 KHz) for 20 minutes, filtered, evaporated the filtrate, the residue were dissolved with 2 mL of chloroform, mixed with 1 g of column chromatography silica gel (60–100 mesh), volatilized the solvent, and added to the treated silica gel column (petroleum ether column, 60–100 mesh, 2 cm × 9 cm), eluted with 150 mL of petroleum ether/ethyl acetate (7 : 3), the eluent was collected, evaporated, an appropriate amount of chloroform was added to dissolve the residue, quantitatively put into a 5 mL measuring bottle, accurately measured 150 *μ*L, operated according to the law from “volatilizing the solvent” under the preparation of the standard curve, and the absorbance was measured and the concentration of total sterols in the test solution was calculated with the standard curve.

### 2.4. Animals and Treatment

Wistar rats weighing 200–230 g were obtained from Changchun Yisi Experimental Animal Technology Co. Ltd. (Changchun, China), Kunming mice weighing 18–22 g were purchased from Changchun Institute of Biological Products Co. Ltd. (Changchun, China), and SD rats weighing 200–230 g were supplied from Liaoning Changsheng Biotechnology Co. Ltd. (Benxi, China). The animal feeding laboratory was kept at 23 ± 2°C and 50 ± 5% relative humidity with a 12 h light/dark cycle. Animals were freely accessed to a commercial standard rat/mouse cube diet and water ad libitum, and they were allowed for acclimation under climate-controlled conditions for a week before use. All studies were conducted in compliance with Good Laboratory Practice Regulations from the State Food and Drug Administration of China. All animal protocols were approved by the Animal Welfare Ethics Committee of Jilin Provincial Academy of Chinese Medicine Sciences [11, 12].

### 2.5. Pharmacodynamic Experiment In Vivo

#### 2.5.1. Effect on Hepatic Fibrosis of Complex Factors in Rats

Wistar rats were randomly divided into two groups, 15 rats were normal control group and the rest 81 rats were model group. Liver fibrosis was induced in rats of model group by following complex factors: rats were fed with high-lipid and low-protein diets (containing 79.5% corn flour, 20% lard, and 0.5% cholesterol) for two weeks and subsequently with pure corn flour for two weeks, meanwhile, these model group rats were subcutaneously injected with 40% carbon tetrachloride olive oil solution twice a week at 3 mL/kg and gavaged with 30% ethanol every other day (1 mL per rat). Rats of the normal control group were given regular feed and drinking water and injected with corresponding volume of olive oil. Animals are weighed once a week.

One month after the establishment of the model, 10 rats died, and the remaining 71 rats were randomly divided into six groups according to the difference in body weight. Gavage is as follows: namely, the normal control group and the model group rats were given 10 mL/kg 0.5% carboxymethylcellulose sodium water suspension; western medicine positive drug group rats were given 40 mg/kg Silibinin capsules (Tianshili Pharmaceutical Group Co. Ltd., Tianjin, China); Chinese medicine positive drug group rats were given 1.08 g/kg Compound Biejia Ruangan Tablet (CBRT, Inner Mongolia Furui Zhongmeng Pharmaceutical Technology Co. Ltd.); the high-, middle-, and low-dose groups of EPS were given 140, 70, and 35 mg/kg, and the volume of gastric perfusion was 10 mL/kg. During the administration period, except the control group, the other groups' rats were given high-fat and low nutrition feed and gavaged with 30% ethanol every other day (1 mL per rat), and 40% carbon tetrachloride oil solution was injected subcutaneously once a week at 3 mL/kg, the control group was given normal feed and drinking water, and injected with equal volume edible oil; the drug was given once a day for 8 weeks [13–15].

After the last administration, the rats were fasted for 16 hours, weighed, blood samples were collected from abdominal aorta, and hyaluronic acid (HA), laminin (LN), procollagen III (PC III), and collagen IV (Col IV) in the serum were determined according to the enzyme-linked immunosorbent assay; total protein (TP) was determined by Bradford method; albumin (ALB) was determined by bromocresol green colorimetry; alanine transaminase (ALT) and aspartate transaminase (AST) activity were determined by the method of Lai's colorimetry; superoxide dismutase (SOD) activity was determined by hydroxylamine method; malondialdehyde (MDA) content was determined by thiobarbituric acid (TBA) method. The liver was fixed in 10% formalin solution for pathological examination [16–22].

#### 2.5.2. Effect on Carbon Tetrachloride-Induced Acute Liver Injury in Mice

66 Kunming mice were randomly divided into six groups, the first group was normal control group and the second group was model group, which all were given 10 mL/kg 0.5% carboxymethylcellulose sodium water suspension by gavage; the third group was positive drug group, which was given 60 mg/kg Silibinin capsule by gavage; the fourth to the sixth groups were the high-, middle-, and low-dose group of EPS, which were, respectively, given 200, 100, and 50 mg/kg by gavage. The drug was given once a day for 10 days.

One hour after the last administration, except for the normal control group, mice of the other groups were intraperitoneally injected with 0.1% carbon tetrachloride olive oil solution 10 mL/kg, fasted for 16 hours later, blood was collected from the ophthalmic vein, centrifuged at 5000 rpm for 5 minutes. The activity of ALT and AST in the serum of mice was determined by the method of Lai's colorimetry. The liver was fixed in 10% formalin solution for pathological examination [23, 24].

#### 2.5.3. Effect on Alcohol-Induced Acute Liver Injury in Mice

68 Kunming mice were randomly divided into six groups, the first group was the normal control group, which was given 10 mL/kg0.5% carboxymethylcellulose sodium water suspension; the second group was the model group, administrated as above; the third group was the positive drug group, which was given 60 mg/kg Silibinin capsules by gavage; the fourth to the sixth groups were the high-, middle-, and low-dose groups of EPS, which were, respectively, given 200, 100, and 50 mg/kg by gavage. The drug was given once a day for 10 days.

After the last administration, except the normal control group, the mice in other groups were fasted for 12 hours, given 15 mL/kg (40°) Beijing Baijiu by gavage. Six hours later, the blood was collected from the ophthalmic vein and the activity of ALT and AST was measured by the method of Lai's colorimetry. The liver was fixed in 10% formalin solution for pathological examination [25–27].

#### 2.5.4. Effect on D-Galactosamine Hydrochloride-Induced Acute Liver Injury in Rats

60 SD rats were randomly divided into six groups, the first group was normal control group and the second group was model group, which all were given 10 mL/kg 0.5% carboxymethylcellulose sodium water suspension by gavage; the third group was positive drug group, which was given 40 mg/kg Silibinin capsules by gavage; the fourth to the sixth groups were high-, middle-, and low-dose groups of EPS, which were given 140, 70, and 35 mg/kg by gavage. The drug was given once a day for 14 days [28–30].

Except for the normal control group, the rats in the other groups were intraperitoneally injected with 400 mg/kg D-galactosamine hydrochloride one hour after the eleventh administration, and again intraperitoneally injected repeatedly with 400 mg/kg D-galactosamine hydrochloride 48 hours later, and blood was collected from the abdominal aorta and centrifuged at 3000 rpm for 10 minutes. ALT, AST, SOD activities, and MDA content in the serum were measured, and the liver was fixed in 10% formalin solution for pathological examination [21–23].

#### 2.5.5. Histopathological Observations

The liver specimens were fixed with 10% neutral formalin and embedded in paraffin. The paraffin-embedded liver tissues were sliced into 4 *μ*m pieces and stained with hematoxylin-eosin (H-E) and Masson-Trichrome (M-T), respectively, for photomicroscopic assessment. A numerical scoring system for histologically assessing the extent of fibrosis was adapted, according to the prevention and treatment plan for viral hepatitis, with minor modifications [10–25]. Briefly, live fibrosis was staged as follows: stage 0, no fibrosis; stage S1, enlargement of the portal area, mild fibrosis; stage S2, fibrosis around the portal area, formation of fibrous septum, preservation of lobular structure; stage S3, fibrous septum with lobular structure disorder, no cirrhosis; stage S4, early cirrhosis.

Additionally, for liver injury induced by various factors (mice induced by carbon tetrachloride and alcohol, and SD rats with acute liver injury caused by D-galactosamine hydrochloride), hepatocyte necrosis or degeneration severity was also graded as follows: grade 0: no hepatocyte degeneration; grade 1: mild hepatocyte degeneration, with no more than 1/4 of the hepatocytes denatured; grade 2: moderate hepatocyte degeneration, with no more than 1/2 of the hepatocytes denatured; grade 3: severe hepatocyte degeneration, with no more than 3/4 of the hepatocytes denatured; and grade 4: severe hepatocyte degeneration, liver tissue is almost replaced by denatured hepatocytes.

The liver scoring examination was performed by a pathologist who was blinded to the rats' treatment assignment. Fibrosis and hepatocyte scores were given after the pathologist had examined throughout three different areas in the tissue slide for each rat, the histopathological changes were observed, and the score data were analyzed.

## 3. Results and Discussion

### 3.1. Medication Composition Analysis

The results showed that total lignans, total lactams, and total sterols were 36.7 ± 0.92, 23.98 ± 0.73, and 8.12 ± 0.70 (g/100 g) respectively, and the sum of the three total components is 68.8 ± 0.92 (g/100 g).

### 3.2. Effect on Hepatic Fibrosis of Complex Factors in Rats

#### 3.2.1. Effect on General Conditions of Rats

The rats in the normal control group had increased body weight, tight fur, luster, free movement, and no death. After subcutaneous injection of carbon tetrachloride, the rats in the model group had reduced activity, rough hair, obvious weight loss, and hair removal. From the second week after modeling, some rats had orange urine, some rats had blood in their urine, there were secretions in their eyes, and some rats lost weight and died. The anatomy of the dead rats showed that there was exudate in the abdominal cavity, small granular protrusions in the liver, the section was thick, the color was yellow or white, and the color of the kidney became dark and dull. Compared with the rats in the model group, most rats in the high-, medium-, and low-dose groups of EPS increased activity, increased weight, and reduced hair withering and depilation.

#### 3.2.2. Effect on Serum Index in Rats with Liver Fibrosis

The contents of LN, HA, COL IV, and PC III in serum of model group were significantly increased compared with the normal control group (*P* < 0.05 or *P* < 0.01), the contents of HA and COL IV were significantly reduced by EPS at doses of the high, middle, and low doses (140, 70, and 35 mg/kg) (*P* < 0.001, *P* < 0.01 or *P* < 0.05), and LN content was significantly reduced by the high and middle doses (*P* < 0.01 or *P* < 0.05). Meanwhile, the content of HA, LN, and COL IV in the Compound Biejia Ruangan Tablets (CBRT) group and Silibinin capsules group was significantly decreased compared with the model group (*P* < 0.001, *P* < 0.01, and *P* < 0.05).

Compared with the model group, the high and middle doses of EPS can significantly reduce the ALT and AST activities (*P* < 0.01, *P* < 0.05) and significantly increase the ALB content (*P* < 0.05). The activities of ALT and AST in Silibinin capsules group and CBRT group were significantly lower and that of ALB were significantly higher than those in the model group (*P* < 0.05).

Compared with the model group, the high-dose group of EPS can significantly improve SOD activity (*P* < 0.05) and significantly reduce MDA content (*P* < 0.05), and the high- and middle-dose group can significantly reduce Hyp content (*P* < 0.05). The content of Hyp in CBRT group and Silibinin capsule group was decreased significantly compared with the model group (*P* < 0.05). For the contents of PC III, EPS and positive drug have no effects.

**Figure 1:**
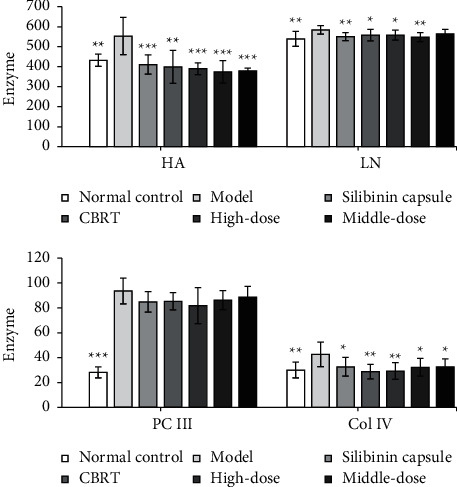
The effect of EPS on the four serum markers of rats with liver fibrosis caused by complex factors (values are mean ± SEM, *n* = 10, compared with model group. *P* values: ^*∗*^＜0.05, ^*∗∗*^＜0.01, ^*∗∗∗*^＜0.001).

**Figure 2:**
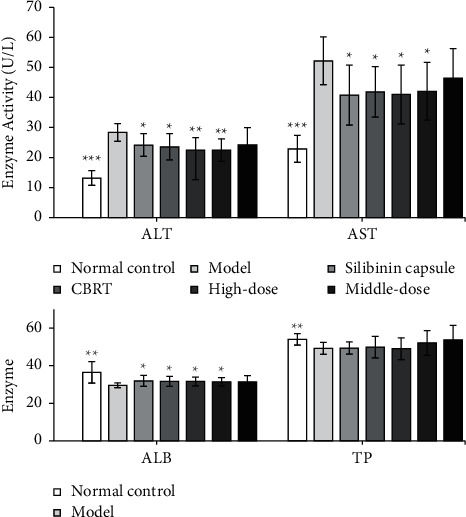
Effect of EPS on liver function index of rats with liver fibrosis caused by complex factors (values are mean ± SEM, *n* = 10, compared with Model group. *P* values: ^*∗*^＜0.05, ^*∗∗*^＜0.01, ^*∗∗∗*^＜0.001).

**Figure 3:**
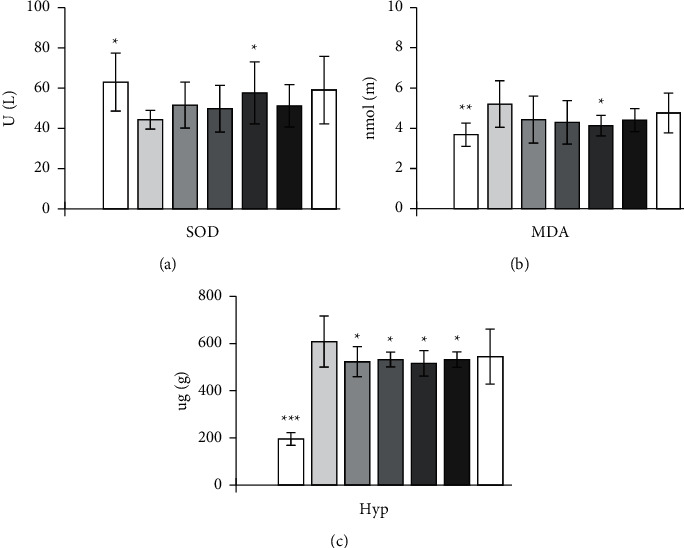
Effect of EPS on free radicals in serum and hydroxyproline in liver of rats with liver fibrosis caused by complex factors (values are mean ± SEM, *n* = 10. compared with model group. *P* values: ^*∗*^＜0.05, ^*∗∗*^＜0.01, ^*∗∗∗*^＜0.001).

The results are shown in Figures 1–3.

#### 3.2.3. Histopathological Evaluation

**Figure 4:**
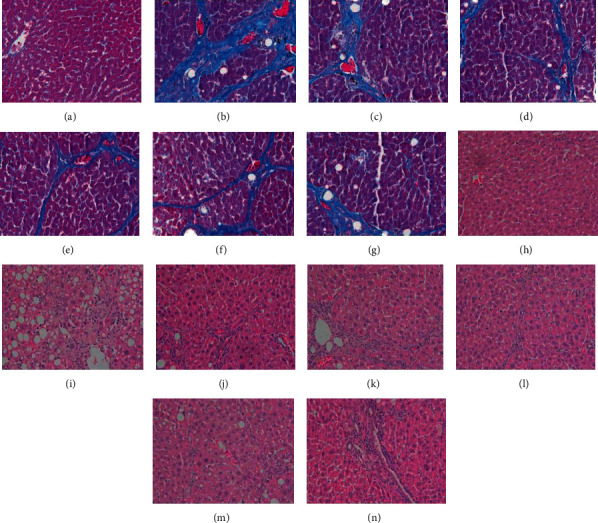
Pathological photos of EPS on liver fibrosis in rats caused by complex factors (magnification, ×400, blue part represent collagen deposition). (a)–(g): Masson staining; (h)–(n): HE staining; (a), (h) control group; (b), (i) model group; (c), (j) Silibinin capsule group; (d), (k): CBRT group; (e), (l) high-dose group of EPS; (f), (m) middle-dose group of EPS; (g), (n) low-dose group of EPS.

According to the prevention and treatment plan for viral hepatitis [10], the histopathological changes were observed and analyzed. The normal control group had normal liver tissue structure, clear portal area and hepatic sinus structure, no fibrous connective tissue hyperplasia, no abnormality, and no inflammatory cell infiltration. No blue-stained collagen fibers separated normal liver tissue to form pseudolobular structure, and the collagen tissue components of blood vessels were dyed blue. In the model group, the proliferation of hepatic fibrous tissue was extensive (especially in the portal area), accompanied by proliferation in the hepatic lobules. In the model group, the hepatic lobules were divided and wrapped by the blue-stained fibrous components of the liver to form pseudolobules of different sizes, degeneration of hepatocytes in pseudolobules, disordered arrangement of hepatocytes, compression of hepatic sinuses, deviation or absence of central veins, and focal lymphocyte infiltration between proliferating fibers. In EPS high- and middle-dose groups and CBRT group, the results showed that the formation of liver fibers and pseudolobules was significantly reduced, which indicated that the formation of liver fibers in rats could be significantly inhibited. The results are shown in Figure 4 and Table 1.

### 3.3. Effect on Carbon Tetrachloride-Induced Acute Liver Injury in Mice

#### 3.3.1. Effect on Liver Function

**Figure 5:**
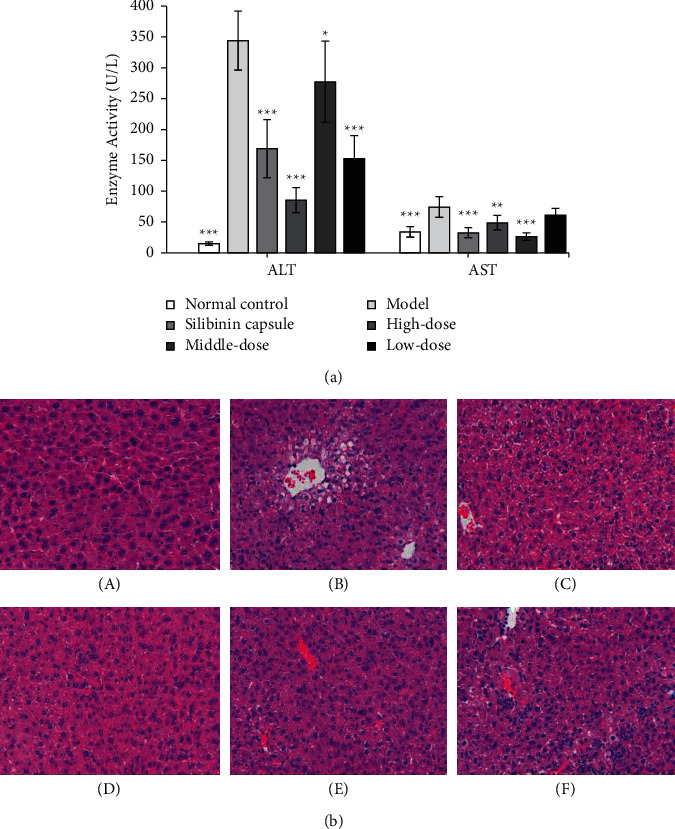
Enzyme activities and histological image effect of EPS on acute liver injury induced by carbon tetrachloride in mice. (a) Enzyme (values are mean ± SEM, *n* = 10. Compared with model group, *P* values: ^*∗*^＜0.05, ^*∗∗*^＜0.01, ^*∗∗∗*^＜0.001). (b) Histological image of liver tissues harvested from mice (HE staining, ×400); (A) control group; (B) model group; (C) Silibinin group; (D) high-dose group of EPS; (E) middle-dose group of EPS; (F) low-dose group of EPS.

The activity of ALT and AST in the model group was significantly higher than that in the normal control group, indicating that the model was established (*P* < 0.001 or *P* < 0.01). The ALT activity of mice with acute liver injury induced by carbon tetrachloride was significantly inhibited in the high-, middle-, and low-dose group of EPS (*P* < 0.001 or *P* < 0.05). The AST activity of mice with acute liver injury induced by carbon tetrachloride was significantly inhibited in the high- and middle-dose groups of EPS (*P* < 0.01 or *P* < 0.001). The activities of ALT and AST in Silibinin capsule group were significantly inhibited compared with the model group (*P* < 0.001). The results are shown in Figure 5.

#### 3.3.2. Pathological Examination Results

The results showed that the control group liver was reddish brown in appearance, soft and elastic in texture, clear in structure of liver lobule, and intact in structure of liver cell, without necrosis, swelling, and other lesions. In the model group, the structure of liver lobule was damaged, inflammatory cell infiltration, spot necrosis, and cell degeneration were observed. Compared with the model group, EPS administration group was alleviated in varying degrees. The results are shown in Figure 5 and Table 2.

### 3.4. Effect on Alcohol-Induced Acute Liver Injury in Mice

#### 3.4.1. Effect on Liver Function

**Figure 6:**
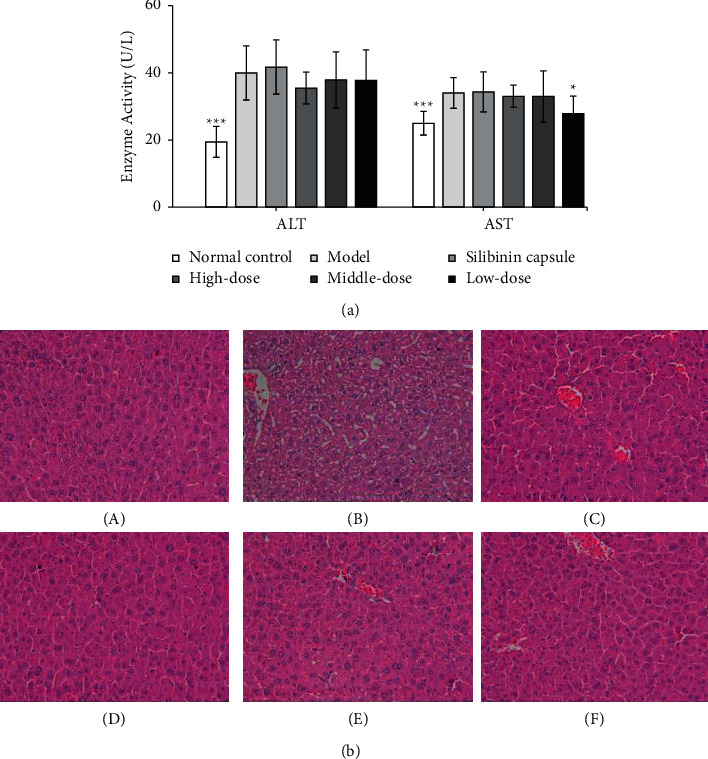
Enzyme activities and histological image effect of EPS on alcohol-induced acute liver injury in mice. (a) Enzyme (values are mean ± SEM, n = 10, compared with model group, *P* values: ^*∗*^＜0.05, ^*∗∗*^＜0.01,^*∗∗∗*^＜0.001). (b) Histological image of the effect of EPS on alcohol-induced acute liver injury in mice (HE staining, ×400) (A) control group; (B) model group; (C) Silibinin group; (D) high-dose group of EPS; (E) middle-dose group of EPS; (F) low-dose group of EPS.

Six hours after intragastric administration of alcohol, the activities of ALT and AST in the serum of the model group were significantly higher than that of the normal control group (*P* < 0.01 or *P* < 0.05), indicating that the model was established. Three dose group of EPS had no significant effect on ALT activity in serum of mice with alcoholism; high-dose group could significantly reduce AST activity compared with the model group (*P* < 0.05). There was no significant effect of Silibinin capsule on ALT and AST activities in serum of mice with alcoholism. The results are shown in Figure 6.

#### 3.4.2. Pathological Examination Results

In the normal group, there was no degeneration of hepatocytes in the liver, the structure of hepatic lobules was clear, and the hepatocytes were arranged in a radial manner from the central vein to the periphery of the lobules. Hepatocyte degeneration with different number of cases was observed in the liver of the model group and each administration group. EPS group can alleviate the degeneration process of liver injury induced by alcohol in mice to varying degrees, and the results are shown in Table 3 and Figure 6.

### 3.5. Effect on D-Galactosamine Hydrochloride-Induced Acute Liver Injury in Rats

#### 3.5.1. Effect on Liver Function

**Figure 7:**
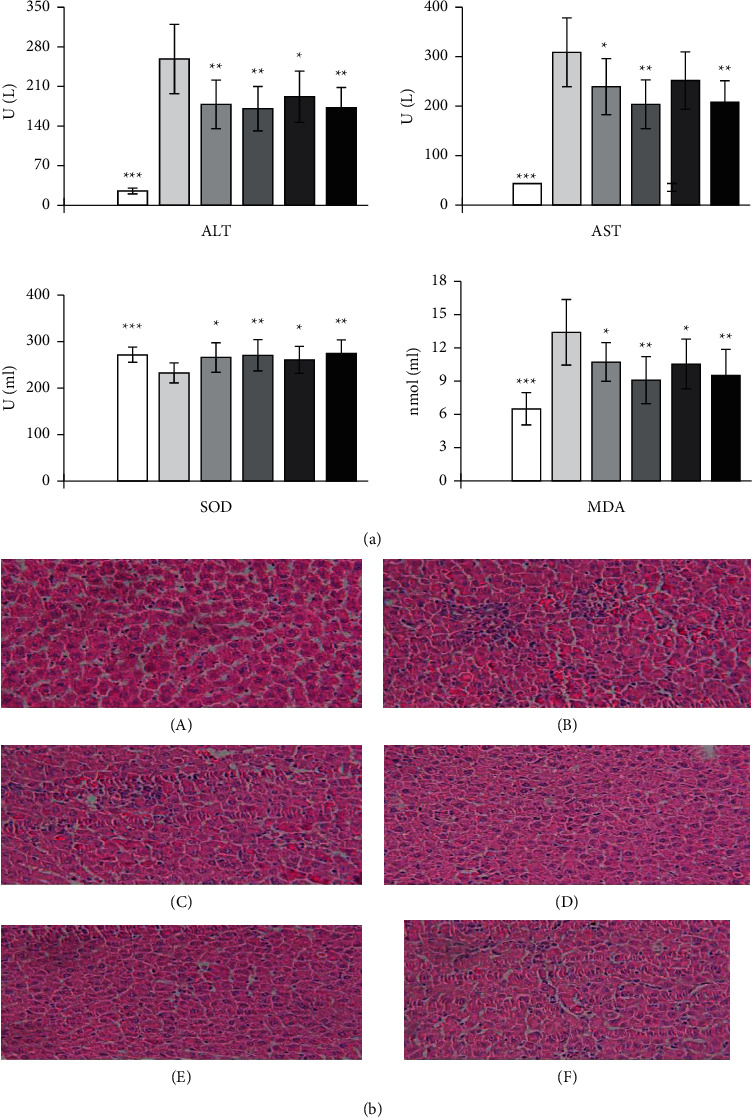
Enzyme activities and histological image of the effect of EPS on liver injury induced by D-galactosamine hydrochloride in rats. (a) Enzyme (values are mean ± SEM, n = 10, compared with model group, *P* values:^*∗*^＜0.05, ^*∗∗*^＜0.01, ^*∗∗∗*^＜0.001). (b) Histological image of pathological photos of the effect of EPS on liver injury induced by D-galactosamine hydrochloride (HE staining, ×400). (A) control group; (B) model group; (C) Silibinin group; (D) high-dose group of EPS; (E) middle-dose group of EPS; (F) low-dose group of EPS.

After D-galactosamine hydrochloride was injected into the abdominal cavity of the model group, the activities or content of ALT, AST, and MDA in the serum of the rats increased significantly compared with the control group (*P* < 0.001), and the activity of SOD decreased significantly, compared with the control group (*P* < 0.001), indicating that the model was established. Compared with the model group, the ALT, AST, and MDA content was significantly decreased and SOD content was significantly increased in the high-dose group (*P* < 0.01); The middle-dose group of EPS can significantly reduce the ALT and MDA activities and improve the SOD activity (*P* < 0.05); the low-dose group of EPS can significantly reduce the ALT and AST activities and MDA content and improve the SOD activity (*P* < 0.01). Compared with the model group, Silibinin capsule group can significantly reduce ALT, AST, and MDA content and significantly improve SOD activity (*P* < 0.01 or *P* < 0.05). The results are shown in Figure 7.

#### 3.5.2. Pathological Examination Results

Normal control group: hepatocytes are arranged radially with the central vein as the center, the structure of hepatic lobules is complete, and there is no hepatocyte degeneration and inflammatory cell infiltration in intrahepatic portal area. In the model group, the liver showed pathological changes, most of the hepatocytes were swollen, the cytoplasm was loose and ballooned, and extensive hepatocyte degeneration and necrosis could be seen; inflammatory cell infiltration was also observed. 5/10 animals showed severe liver injury and degeneration of a large area of hepatocytes. The number and degree of liver degeneration in the positive western medicine group decreased significantly. The number and degree of liver degeneration of animals in EPS administration groups were significantly reduced. Comparative analysis shows that EPS can effectively alleviate the liver injury of rats caused by D-galactosamine. The results are shown in Figure 7 and Table 4.

## 4. Discussion and Conclusion

Liver fibrosis occurs as a result of many chronic liver diseases such as hepatitis viral infection, including hepatitis B and C, in patients. Liver fibrosis plays a pivotal role in liver function impairment. It is characterized by altered hepatic function and the excessive accumulation of extracellular matrix proteins including collagen that occurs in most types of chronic liver diseases. The progression of liver fibrosis often develops into irreversible cirrhosis and is associated with liver cancer. Therefore, it is urgent to reduce the damage to the liver and to slow down the progress of liver injury to fibrosis and cancer.

Carbon tetrachloride complex liver fibrosis model is a continuous infusion of Chinese Baijiu (liquor) by repeated injection of carbon tetrachloride, feeding high-fat (lard, cholesterol), low-protein (cornmeal) feed, resulting in fatty degeneration, necrosis, and fibrous hyperplasia of liver cells in rats. The model is stable, reliable, and has a high success rate. It is a commonly used animal model of liver fibrosis. Rats were administered EPS by gavage for 8 weeks, which can significantly reduce the levels of hyaluronic acid (HA), laminin (LN), type IV collagen (Col IV) content, and malondialdehyde content and inhibit the increase of alanine aminotransferase (ALT) and aspartate aminotransferase (AST) activities in serum. The content of serum albumin (ALB) was significantly increased, the activity of superoxide dismutase (SOD) increased, and the content of hydroxyproline (Hyp) significantly reduced in liver tissue. Liver histopathology showed that EPS could significantly reduce the formation of liver fibrosis and inflammatory cell infiltration in rats, indicating that EPS could reduce the pathological damage of compound factors to rat liver tissue.

The mechanism of EPS in the treatment of liver fibrosis was as follows:

① Protect hepatocytes, prevent the further development of liver injury, and promote the recovery of liver function.
② Inhibit the release of cytokines and promote the repair of damaged cells.
③ Scavenge oxygen free radicals and resist lipid peroxidation damage.
④ Inhibit the formation of ECM and promote the degradation of pathological deposited collagen.

Carbon tetrachloride is the most commonly used and classic model of acute liver injury in mice induced by chemical drugs. After entering hepatocytes, carbon tetrachloride dissolves the lipids of mitochondrial membrane, thus affecting the structure and function of mitochondria, reducing the synthesis of enzyme proteins, causing the destruction of enzymes, thus affecting the metabolic function and energy generation, and denaturing and necrotizing hepatocytes. During hepatocytes degeneration and necrosis, the permeability of cell membrane increases, and a large amount of ALT and AST flows into the blood, which increases the serum ALT and AST. Therefore, the determination of the content of ALT and AST in serum can reflect the degree of hepatocyte degeneration and necrosis. EPS can significantly inhibit the activities of ALT and AST in serum of mice with acute liver injury induced by carbon tetrachloride and significantly reduce the degree of liver pathological injury, indicating that it has a protective effect on acute liver injury induced by carbon tetrachloride.

Alcohol dependence and alcohol abuse have become an important social and medical problem in modern society. The liver is the main organ of alcohol metabolism *in vivo*. Excessive alcohol intake will lead to different degrees of damage to the liver and abnormal increase of serum ALT and AST. The high-dose EPS group can significantly reduce the activity of AST in serum of alcoholic mice and reduce the degree of pathological damage of damaged liver, indicating that it had a certain protective effect on acute liver injury caused by alcohol.

D-galactosamine is currently recognized as a relatively good experimental animal model for studying the pathogenesis of viral hepatitis and observing the curative effect of drugs. Its poisoning mechanism is that D-galactosamine enters the body and combines with uridine phosphate (UDP) to form UDP galactosamine, resulting in the depletion of uridine phosphate, so that the nucleic acids, proteins, and the synthesis of substances such as lipopolysaccharide are inhibited, which limits the generation and supplement of organelles and enzymes. Organelles are damaged, cell membranes are damaged, and calcium ions flow into cells, resulting in hepatocyte damage. EPS can significantly reduce the activity of ALT and AST and the content of MDA in serum of D-galactosamine hydrochloride-induced liver injury model rats, increase the activity of SOD, and reduce the pathological damage of liver tissue, indicating that EPS had a protective effect on D-galactosamine-induced liver injury in rats.

In conclusion, EPS can effectively inhibit the formation of liver fibrosis and has obvious preventive and therapeutic effects on liver injury caused by various factors. In summary, our findings showed that EPS exhibits potent activities and should be considered a good option and an additional source of natural compounds for the treatment of hepatic fibrosis and hepatic injury. EPS is a promising drug candidate [26–37].

## Figures and Tables

**Table 1:** Pathological score of EPS on liver fibrosis in rats caused by complex factors.

Group	Number of animals	Stages of liver fibrosis					*P*
		0	S1	S2	S3	S4	
Normal control group	14	14	0	0	0	0	
Model group	13	0	0	0	1	12	
Silibinin capsule	11	0	0	1	4	6	0.119
CBRT group	10	0	1	1	4	4	0.030
High dose of EPS	12	0	1	2	4	5	0.026
Middle dose of EPS	11	0	0	2	3	6	0.106
Low dose of EPS	14	0	0	3	3	8	0.105

**Table 2:** Pathological scores of EPS on the acute liver injury induced by carbon tetrachloride in mice.

Group	Number of animals	Classification of liver injury				*P*
		0	1	2	3	
Normal control group	10	10	0	0	0	≤0.001
Model group	12	0	2	5	5	
Silibinin capsule	11	1	6	3	1	0.023
High dose of EPS	11	2	7	2	0	0.001
Middle dose of EPS	11	0	6	4	1	0.044
Low dose of EPS	11	0	5	4	2	0.151

**Table 3:** Pathological score of EPS on alcohol-induced acute liver injury in mice.

Group	Number of animals	Classification of liver injury				*P*
		0	1	2	3	
Normal control group	10	10	0	0	0	0.01
Model group	11	2	5	1	3	
Silibinin capsule	12	3	9	0	0	0.169
High dose of EPS	12	5	6	1	0	0.104
Middle dose of EPS	11	2	6	3	0	0.562
Low dose of EPS	11	3	8	0	0	0.171

**Table 4:** Comparative statistical results of liver injury ratings of rats in each group.

Group	Number of animals	Classification of liver injury					*P*
		0	1	2	3	4	
Normal control group	10	9	1	0	0	0	≤0.001
Model group	10	0	0	2	3	5	
Silibinin capsule	10	1	1	2	2	4	0.436
High dose of EPS	10	2	2	3	2	1	0.011
Middle dose of EPS	10	1	2	2	2	3	0.165
Low dose of EPS	10	1	1	2	3	3	0.280

## Data Availability

The data are included in the manuscript.
